# An Unexpected Reaction to Topical Garlic Medicament – A Case Report of Irritant Contact Dermatitis Successfully Managed in Primary Care

**DOI:** 10.7759/cureus.33657

**Published:** 2023-01-11

**Authors:** Jazlan Jamaluddin, Siti Nuradliah Jamil

**Affiliations:** 1 Family Medicine, Klinik Kesihatan Sauk, Kuala Kangsar, MYS; 2 Family Medicine, Klinik Kesihatan Pasir Gudang, Johor Bahru, MYS

**Keywords:** complementary and alternative medicines, primary care, irritant, garlic, contact dermatitis

## Abstract

Irritant contact dermatitis (ICD) is a common skin condition in primary care. The frequent cause of ICD includes hair dye, nail polish, paints, cleaners, soap, and detergent. We present a case of ICD caused by topical garlic medicament, successfully identified and managed in primary care. A 20-year-old woman presented with a sudden onset of multiple painful localized blisters on the right antecubital fossa. She reported applying raw garlic paste to the area one day before the clinic visit to treat mild itchiness. She had no known allergies or medical illnesses. Otherwise, there were no rashes elsewhere or oral and genital ulcers. She was not on any regular medication or taking traditional medication. Examination revealed multiple bullae on the antecubital fossa with perilesional erythema. The lesion was sharply bordered within the contact areas, was asymmetric, and did not spread elsewhere. The clinical history of immediate bullae formation after direct contact with garlic was consistent with ICD due to garlic medicament. The lesions were managed with regular dressings. At one week follow-up, the lesions had healed well. She was advised to avoid further application of topical garlic medicines. Although Allium sativum (garlic) has been used either topical or orally as a medicinal treatment worldwide for thousands of years to treat various conditions, it still has the potential to cause irritant dermatitis when applied to skin and mucosa. Patients and healthcare providers should be cautious of the potential side effects, such as ICD, when using garlic for medicinal purposes.

## Introduction

Irritant contact dermatitis (ICD) is a frequently encountered skin condition in primary care. It is caused by direct contact with an irritant to the skin epidermis, which triggers the immune system resulting in an inflammatory response [1]. ICDs are influenced by endogenous and exogenous factors [2]. Endogenous factors include age, sex, region of skin, and the presence of atopy and others. Exogenous factors include the type of irritant, concentration of exposure, duration, repetition, and others. Acute ICD usually causes bright erythema, vesiculation, and weeping, which may mimic a chemical burn or sunburn. The causes of ICD can often be identified based on the region of skin involvement [1]. For upper limb involvement of ICD, the most common cause includes soap, detergent, solvents, and other occupational exposure to irritants. This report presents a case of ICD caused by topical garlic medicament, successfully identified and managed in primary care.

Allium sativum (garlic) has been used as a medicinal treatment worldwide, especially in Asia and Eastern Europe, for thousands of years, either by topical use or orally to treat various conditions [3]. Much research has been done to study its effects on cancer prevention and immunomodulatory and antioxidant properties. Clinical data have shown garlic to be effective in treating and preventing hypertension, hyperlipidemia, and even atherosclerotic vascular changes. However, garlic has been shown to cause various adverse effects, such as odor, gastrointestinal symptoms, hypocoagulation, and allergies [4]. Allergic reactions to garlic are usually manifested as allergic contact dermatitis (ACD), photoallergy, generalized urticaria, angioedema, pemphigus, and anaphylactic reaction. Garlic contains sulfur compounds, the most important being diallyl thiosulfinate (Allicin), with anti‐inflammatory and immunomodulatory effects. However, Allicin can result in acantolysis and coagulative necrosis of the epidermis, compromising the integrity of the skin barrier and causing ICD [5].

This article was previously presented as a poster at the 2022 International Conference on Post-COVID Healthcare, Medical Research and Education from March 29 to 31, 2022 [6].

## Case presentation

**Figure 1:**
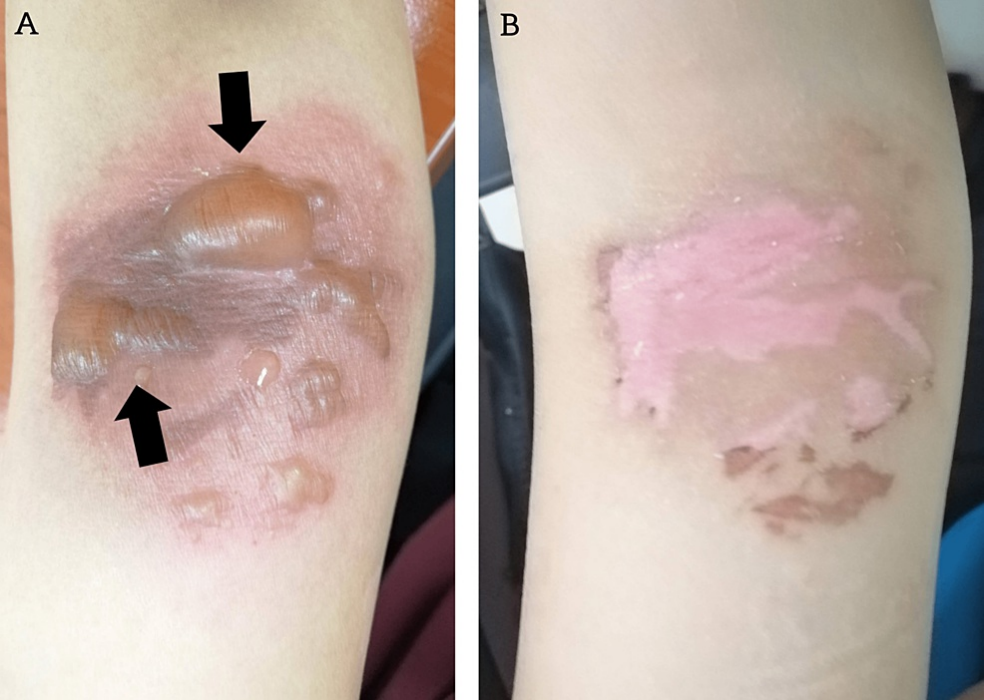
Multiple bullae on the antecubital fossa with perilesional erythema (Panel A). The lesions resolved after one week (Panel B) with regular dressing.

A 20-year-old woman presented with multiple painful blisters with redness on the right antecubital fossa. Otherwise, she had no other symptoms. One day before the presentation, she reported applying crushed raw garlic paste made by her mother to the area to treat mild itchiness due to skin dryness. There was no bite mark, other rashes elsewhere, or oral and genital ulcer. She was not taking any medication, vitamins, or supplements. She reported no known allergies or medical illnesses. Examination revealed multiple bullae on the right antecubital fossa with perilesional erythema (Figure 1). The lesion was sharply bordered within the contact areas, was asymmetric, and did not spread elsewhere. The clinical history of immediate bullae formation after direct contact with garlic was consistent with ICD due to garlic medicament.

The lesions were managed with isotonic saline gauze dressings. A tablet of paracetamol of 1 gm three times daily was prescribed for pain control. At one week follow-up, the lesions had healed well. She was advised to avoid further application of topical garlic medicines.

## Discussion

This case describes a young, healthy woman with sudden-onset ICD after topical garlic exposure. Previous cases of ICD after garlic exposure have been reported; however, past cases typically describe the ICD of the fingers in individuals who cook with garlic [4]. In contrast, the current case describes the ICD of the antecubital region, which is somewhat unusual. In addition, bullae are uncommon after garlic exposure [7], as in the current case.

Erythema, blisters, pustules, hemorrhage, crusts, scales, erosions, and pruritus or pain typically characterize contact dermatitis. Clinically, acute ICD can be distinguished from ACD by its rapid onset [8]. Although ICD and ACD can occur anywhere, the lesions of ICD typically do not spread distantly. On the other hand, ACD usually develops in several different clinical phases. The first phase, the erythematous phase, is characterized by an unclear margin of erythema or skin edema. The' madidans' second phase is characterized by erosions and moistening. The third phase starts when crusts appear, and the final or squamous phase starts when the horny layer repairs itself. The lesions of ACD may be similar to ICD initially, but they often spread to other areas later. ICD and ACD can develop into chronic phases with lichenification and fissures if not treated early.

Similar to ICD of other causes, garlic‐induced skin lesions are influenced by the type of preparation (paste, cream, oil, with alcohol or crushed pieces of raw garlic), prior use, type of dressing (occlusive or non‐occlusive), the presence of other skin disorders (such as eczema), exposure time and predisposition. In the clinical setting, an allergic reaction to garlic can be distinguished from ICD by a positive type‐IV patch test reaction for diallyl disulfide [1,5]. However, a history of immediate skin lesions formation after direct contact with specific irritants is often adequate to confirm the cause of ICD. Acute ICD usually involves appropriate dressing and symptomatic treatment [1]. It usually resolves after about one to two weeks, avoiding irritants, as in this case. However, complications may still occur that may require wound debridement and even skin transplant [3]. Although garlic has been used for generations to treat various skin lesions, the risks of these home remedies must be highlighted.

## Conclusions

This case highlights a young previously-healthy woman who developed an ICD after applying garlic to her skin. Although Allium sativum (garlic) has been used either topical or orally as a medicinal treatment worldwide for thousands of years to treat various conditions, it still has the potential to cause ICD when applied to skin and mucosa. Patients and healthcare providers should be cautious about the potential side effects of using garlic for medicinal purposes, such as the ICD. Patients with a history of ICD should also be vigilant and avoid this allergen.
